# Monoclonal antibody anti-PBP2a protects mice against MRSA (methicillin-resistant *Staphylococcus aureus*) infections

**DOI:** 10.1371/journal.pone.0225752

**Published:** 2019-11-27

**Authors:** Felipe Betoni Saraiva, Ana Caroline Cavalcante de Araújo, Anna Érika Vieira de Araújo, José Procópio Moreno Senna

**Affiliations:** Instituto de Tecnologia em Imunobiológicos – BioManguinhos – FIOCRUZ, Rio de Janeiro, Brazil; Instituto Butantan, BRAZIL

## Abstract

Methicillin-resistant *Staphylococcus aureus* (MRSA) is a multidrug-resistant bacterium responsible for serious nosocomial and community-acquired infections worldwide. Since few antibiotics are effective for treating MRSA infections, the development of new therapies is of great importance. Previous studies demonstrated that PBP2a is a target that generates protective antibodies against MRSA. A murine monoclonal antibody (MAb) that recognizes PBP2a from MRSA strains was previously isolated and characterized. In this report, we evaluated the biodistribution of this MAb in blood and tissues, as well as the extent of protection conferred using prophylactic and therapeutic assays compared to vancomycin treatment. Biodistribution was evaluated 12–96 h after MAb administration. It predominantly remained in the serum, but it was also detectable in the kidneys, lungs, and spleen at low concentrations (about 4.5% in the kidneys, 1.9% in the lungs, and 0.7% the spleen) at all observed timepoints. Prophylactic studies in a murine model demonstrated a significant bacterial load reduction in the kidneys of the groups treated with either with IgG (greater than 3 logs) or F(ab’)_2_ (98%) when compared to that of the control groups (untreated). Mice were challenged with a lethal dose, and the survival rate was higher in the treated mice. Treatment with the MAb resulted in a bacterial load reduction in the kidneys similar to that of mice treated with vancomycin, and a MAb/vancomycin combination therapy was also effective. These results demonstrate that an anti-PBP2a MAb may be a promising therapeutic for treating MRSA infections.

## Introduction

The emergence of infections caused by multidrug-resistant (MDR) bacteria is increasing at an alarming rate. A study conducted by O’Neill indicates that the number of deaths caused by antimicrobial-resistant bacteria could reach 10 million in 2050 [1]. Methicillin-resistant *Staphylococcus aureus* (MRSA) is an MDR bacterium responsible for serious infections in communities and hospitalized patients worldwide. This pathogen is resistant to all β-lactams and various other classes of antibiotics. Ensuring adequate and effective treatment has become a complex problem. The resistance of MRSA strains to β-lactams is due to the presence of PBP2a, a transpeptidase enzyme that exhibits low affinity to this class of antibiotics [2]. Glycopeptides are considered the last resource for treatment of MRSA infections; however, reports of vancomycin-intermediate *S*. *aureus* (VISA) and vancomycin-resistant *S*. *aureus* suggest that these bacteria could soon become resistant to all currently available antibiotics [3]. In the absence of new antibiotics that effectively treat MRSA infections, new approaches are considered to be of high importance. Passive immunotherapy (serum therapy), which was used to treat bacterial infections at the end of the 19th century, was replaced by antibiotics. However, the discovery of monoclonal antibodies (MAbs) 70 years ago established new approaches for the treatment of cancer, in addition to autoimmune and infectious diseases using immunotherapy [4]. Currently, MAbs are being investigated by various research groups for the treatment of bacterial infections, including those caused by *S*. *aureus*, *Pseudomonas aeruginosa*, *Bacillus anthracis*, *Clostridium difficile*, and *Escherichia coli* [5].

Numerous targets of *S*. *aureus* have been tested for use in immunotherapies, including lipoteichoic acid [6], alpha toxin [7], fibrinogen binding protein [8], and protein A [9]. However, none of these targets have been approved for clinical use. PBP2a, a multi-modular class B penicillin-binding protein (PBP), is located external to the membrane of all MRSA strains [10]. Therefore, PBP could be accessible to the host’s immune system. Two studies employing DNA vaccination demonstrated that PBP2a generates an immune response and eliminates bacteria after systemic infection in immunized mice [11–12]. We recently reported the generation and characterization of murine MAbs that specifically bind PBP2a *in vitro* with high affinity [13]. In another study, we demonstrated that this anti-PBP2a antibody recognizes PBP2a protein on the bacterial surface using an immunofluorescence assay [14]. Here, we demonstrate that this MAb also confers protection in a murine model administered both prophylactic and therapeutic treatments. The results suggest that MAbs directed against PBP2a are a promising approach for treating infections caused by MRSA strains.

## Materials and methods

### Monoclonal antibody production and purification

MAbs were obtained as previously described [9]. Briefly, hybridoma cells were grown in Dulbecco’s modified eagle medium (DMEM, Life Technologies, Carlsbad, CA) with 10% of Ultra Low IgG Fetal Bovine Serum (Life Technologies, Carlsbad, CA) in 100-mL bottles at 37°C under 5% CO_2_. Supernatants were harvested by centrifugation and passed through a 0.2-mm filter before liquid chromatography purification with MAbselect SuRe^™^ protein A resin (GE Healthcare). Purified antibodies were dialyzed against phosphate-buffered solution (PBS) (10 mM sodium phosphate, 0.15 M sodium chloride, pH 7.4), concentrated with Amicon ultrafiltration units, quantified at 280 nm using a Nanodrop spectrophotometer (Thermo Scientific), and stored at 4°C or in 40% glycerol at –20°C. A SDS-PAGE showing the purified MAb anti-PBP2a is represented in Supporting Information (S1 Fig).

### Bacterial strains

Iberian Clone MRSA was obtained from the Unité des Agents Antibactériens—Institut Pasteur, and the BEC/BMB9393 (Brazilian Epidemic Clone) MRSA strain was obtained from the Instituto de Microbiologia Paulo de Goés—Universidade Federal do Rio de Janeiro. Both were epidemic MRSA strains of different genetic backgrounds and they were characterized by pulsed-field gel electrophoresis (PFGE) and multi-locus sequencing type (MLST) typing (BEC ST239, Iberian MRSA ST247) [15, 16].

### *In vivo* assays

All animal procedures were approved by the Ethics Committee on Animal Research (CEUA), FIOCRUZ (License number LW-30/12). Animals were provided by ICTB/Fiocruz and maintained in closed cages (dimensions: 37 cm in length, 24.2 cm in width, 24 cm in height) in a ventilated Microisolator Rack's system (ALESCO^®^) with autoclaved pine flakes as a substrate. Cage changes were performed weekly. The mice were allocated in groups of four animals per cage and maintained under a 12 h/12 h (day/night) light cycle with unrestricted access to food and filtered water. Inoculations were performed simultaneously in all groups. Inoculation with the antibodies was performed during the first hours of the day cycle, and infection of the animals was performed during the last hours of the day cycle according to established protocols. For the therapeutic assays, infections were performed before the administration of antibodies or vancomycin, as described in the protocol. Using a clinical evaluation based on the ARRIVE guidelines, animals with severe clinical symptoms characterized by suffering or inability to access water or feed (humane endpoint) were euthanized with anesthesia at doses of 200 mg/kg ketamine hydrochloride and 20 mg/kg xilazine hydrochloride administered intramuscularly. After a few minutes of anesthetic administration, and in the absence of reflexes, sodium thiopental was administered intraperitoneally at a dose of 150 mg/kg.

#### Animal baseline data

Mice of varying weights were assigned to each cage so that the sample was heterogeneous within each group. Animal health was assessed by a veterinarian. Sanitary monitoring of the colony was performed quarterly based on a list of pathogens as recommended by the Federation for Laboratory Animal Science Associations (FELASA).

### Determination of lethal and sublethal doses (LD_50_) for MRSA strains

The lethal dose and LD_50_ were determined according to the Reed-Muench method with modifications for the MRSA strains employed in this study [17]. Four groups of 8-week-old female BALB/c mice (n = 4 per group, total of 16 animals) were inoculated intraperitoneally (IP) with bacteria (1x10^8^, 5x10^8^, 1x10^9^, and 5x10^9^ CFU [colony forming unit]) and observed over 7 days. Afterwards, all surviving animals were euthanized according to the approved CEUA protocol.

### Systemic infection and bacterial renal quantification assays (prophylactic assays)

MRSA strains were grown until they reached exponential phase (OD_600_ 0.6). They were then washed and resuspended in sterile PBS at OD_600_ 0.5, which corresponded to approximately 2×10^8^ CFU (a sublethal dose). This concentration was calculated by a 10-fold serial dilution, and bacteria were cultured on Brain Heart Infusion (BHI) agar plates containing 10 μg/mL of oxacillin for 18 h at 37°C.

Three groups of 8-week-old female BALB/c mice (n = 7 per group, total of 21 animals) received an IP dose of either (a) 500 μg (25mg/kg) of purified anti-PBP2a MAb or (b) mock MAb (4G2 –a murine monoclonal antibody against Yellow fever) on the first day. To demonstrate *in vivo* antibody-neutralizing activity, another group (c) was treated with 350 μg (17.5 mg/kg) of anti-PBP2a F(ab’)_2_. These fragments were obtained as previously described in Araujo *et al*. [14].

On the second day, mice were infected with a sublethal dose and observed until the fifth day. On the sixth day, animals were euthanized, and the kidneys were aseptically removed. These organs were then homogenized in 1 mL of sterile Luria Broth (LB), diluted by a 10-fold serial dilution, and plated as previously described. The resulting colonies were counted in order to calculate the concentration of bacteria in the kidneys. During the experiment, mice were monitored daily for clinical symptoms, such as apathy, rough hair coat, or fever. Anorexia and loss of mobility were considered humane endpoints.

### Survival assay

MRSA strains were grown under the same conditions previously described, but an inoculum adjustment was performed for the previously established LD_50_. Eight-week-old female BALB/c mice (n = 10 per group, total of 40 animals) received one IP dose of 500 μg (25 mg/kg) purified MAb on the first day. On the following day, these animals and a control group were inoculated IP with one dose of 3.3x10^8^ CFU (BEC—Brazilian Epidemic Clone MRSA strain), 4.2x10^8^ CFU (Iberian MRSA strain), or PBS (control group). The animals were observed for 10 days, and the survivors were subsequently euthanized.

### Therapeutic assay with vancomycin

Four groups of 8-week-old female BALB/c mice (n = 5 per group, total of 20 animals) received an infective dose (5–7.0×10^6^ CFU [BEC MRSA]). The animals received doses of purified MAb, vancomycin, MAb + vancomycin, or PBS (negative control) as described in the Table 1:

**Table 1:** Description of groups (treated and control) used in the therapeutic assay.

Group	Description
1	MAb, 500 μg (25 mg/kg), administered IP on the first day
2	Vancomycin, 150 μg (7.5 mg/kg), administered by an intramuscular route, 12/12 hours, 3 days (5 doses)
3	Association (MAb + vancomycin) as previously described to groups 1 and 2
4	PBS (negative control), administered IP on the first day

The first dose of MAb and vancomycin was administered 6 h after the administration of the infective dose. Animals were subjected to euthanasia on the fourth day. The animals’ kidneys were aseptically removed and subjected to bacterial quantification according to previously described methods.

#### Biodistribution assay

In order to evaluate the presence of the MAb in serum and different tissues, 9-week-old female BALB/c mice were used in this assay. Mice (n = 18) received IP inoculations of 500 μg (25 mg/kg) of either purified MAb or PBS (negative control). Samples of serum, lungs, spleen, and kidneys were collected at different time points after inoculation (MAb: 12, 24, 48, 72, and 96 h; PBS: 96 h; three mice per time point). Tissues were homogenized as previously described. An enzyme-linked immunosorbent assay (ELISA) was performed as previously described [9] with some adaptations for MAb quantitation. Briefly, plates were coated with 1.0 mg/mL recombinant PBP2a, diluted in 0.1M carbonate/bicarbonate coating buffer (pH 9.6), and incubated overnight at 4°C. The plates were washed four times (PBP 0.05% Tween-20, Sigma-Aldrich) and blocked (PBS containing 5% non-fat milk, for 2 h at 37°C. After, the plates were incubated for a further 2 h at 37°C with serum or homogenized tissues. Subsequently, the plates were washed and incubated at 37°C for 90 minutes with the secondary antibody (anti-mouse polyvalent peroxidase-conjugated; Sigma-Aldrich) diluted 1:30.000. To develop the reaction, plates were washed and incubated 10 min at room temperature with chromogenic substrate solution (TMB peroxidase, Bio-Rad, Hercules, CA). To stop the reaction, 50 mL/well 2M H_2_SO4 was added and the optical density (OD) was read on a microplate reader Benchmark (Bio-Rad) ar 450 nm. For each assay, a standard curve was created using a previously quantified MAb [9] to ensure that neither endogenous antibodies nor other components of the samples would interfere with the analysis. A negative control mouse sample was added to the standard curve with the same volume and dilutions as the analyzed samples. The amount of MAb present in each sample was then calculated by plotting the results on the standard curves.

#### Statistical analysis

All statistics were performed using GraphPad Prism (version 5). For the comparison of bacterial burden, a Mann-Whitney U test was used. Survival was plotted as Kaplan-Meier curves and analyzed using log-rank (Mental-Cox) tests. Differences were considered significant when the *p*-value was <0.05, and these *p*-values are indicated in the figures by an asterisk (*).

## Results

In order to illustrate the chronological sequence of animal assays, a timeline comprising all assays is presented in Supporting Information (S2 Fig).

### Determination of lethal and sublethal doses for MRSA strains

Lethal and sublethal dose determinations were required for the *in vivo* protection assays, and these were evaluated using two models: 1.) bacterial load in the kidneys after challenge with a sublethal dose and 2.) a survival test after systemic infection with a higher bacterial inoculation that caused death in more than 50% of the animals. The IP route was chosen because of the easier method of administration and because it avoided bacterial loss. The COL MRSA strain was also tested; however, it proved to be less virulent, requiring a higher infective dose in relation to the epidemic BEC and Iberian MRSA strains (BEC ST239, Iberian ST247) evaluated. Therefore, it was not used in protection assays. For both MRSA clones, doses of 5.0×10^8^ CFU killed more than 50% of infected mice (n = 2/4), and doses of 1.0×10^8^ CFU generated a bacterial load in the kidneys without mortality.

### Bacterial load in the kidneys after systemic infection

**Fig 1:**
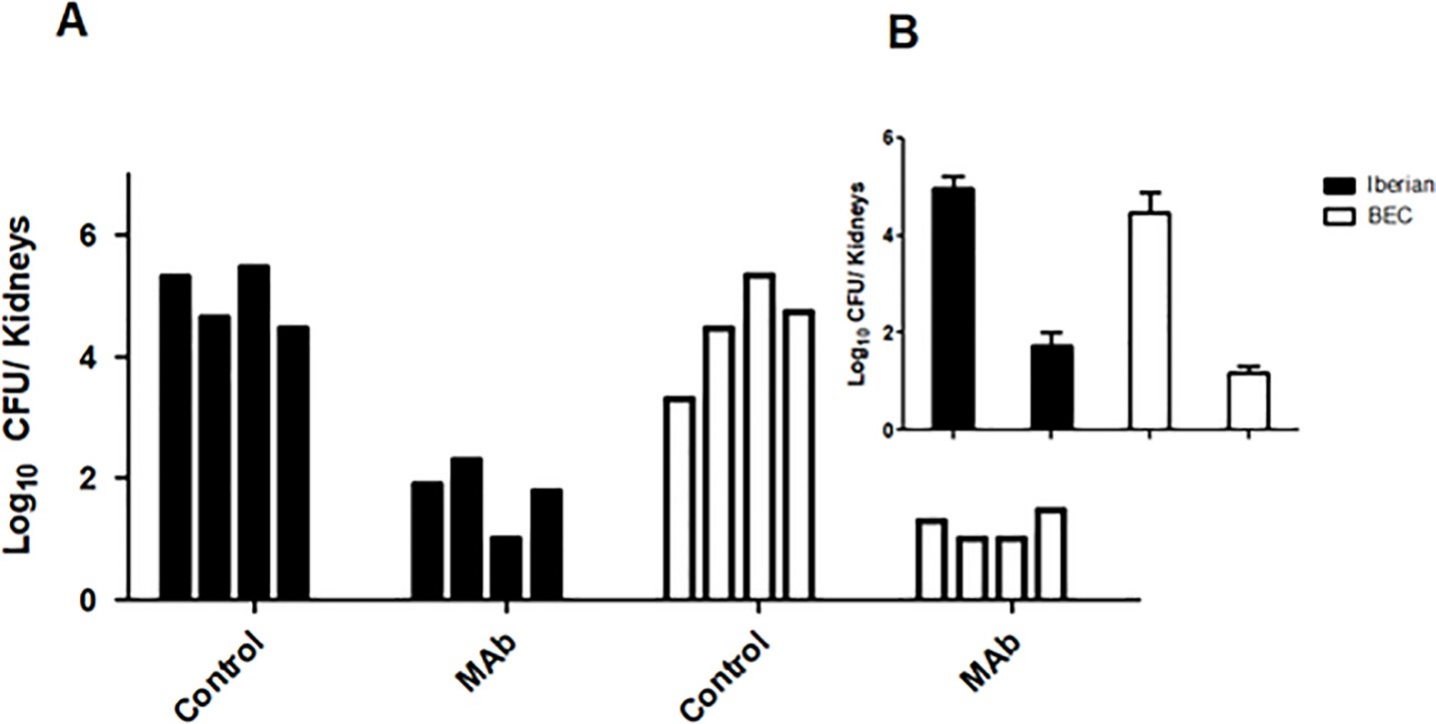
Bacterial load in kidneys after systemic infection with different MRSA strains. Groups (four mice per group) of treated (MAb) and untreated (control) mice were infected via IP injection with 2.4x10^8^ CFU of Iberian (black) or 1.6x10^8^ BEC (white) MRSA clones, and bacterial quantitation was performed after 5 days. **A**. Bacterial load in individual mice: Iberian Control (2.1x10^5^, 4.4x10^4^, 3.1x10^5^, and 2.9x10^4^ CFU); Iberian MAb-treated (8.0x10^1^, 2.0x10^2^, 1.0x10^1^, and 6.0x10^1^ CFU); BEC control (2.0x10^3^, 2.9x10^4^, 2.2x10^5^, and 5.2x10^4^ CFU); BEC MAb-treated (2.0x10^1^, 1.0x10^1^, 1.0x10^1^, and 3.0x10^1^ CFU) (*p<0.05 for Iberian and BEC assays). **B**. Comparative mean for both groups challenged with Iberian and BEC MRSA, respectively (Iberian control: 2.11x10^5^ CFU; Iberian MAb treated: 8.75x10^1^ CFU; BEC control: 7.57x10^4^ CFU; BEC MAb treated: 1.75x10^1^ CFU).

Using a renal quantification model after infection, these assays demonstrated that a prophylactic treatment with anti-PBP2a MAbs reduced the number of bacteria in vital organs (kidneys) after a systemic infection. A reduction of more than 3 logs (1,000 times) was observed in independent assays using two epidemic MRSA strains (Fig 1), which showed similar levels of bacterial reduction (*p*<0.05 for assays with Iberian and BEC MRSA strains). No deaths, clinical signs, or morphological alterations in the kidneys were observed in the treated animals.

In order to demonstrate that *in vivo* protection is due to the antibody binding its target (PBP2a) and eliciting an antibody-neutralizing effect, an anti-PBP2a fragment F(ab’)_2_ was tested. Since these MAb fragments lack the Fc region, they are unable to elicit an immune response to eliminate the pathogen. We used 350 μg of F(ab’)_2_ instead of 500 μg for IgG MAb in order to maintain an equivalent molar concentration between both treated groups (F[ab’]_2_ or IgG) since the molecular mass of IgGs (150 kDa) is greater than F(ab’)_2_ (100 kDa).

**Fig 2:**
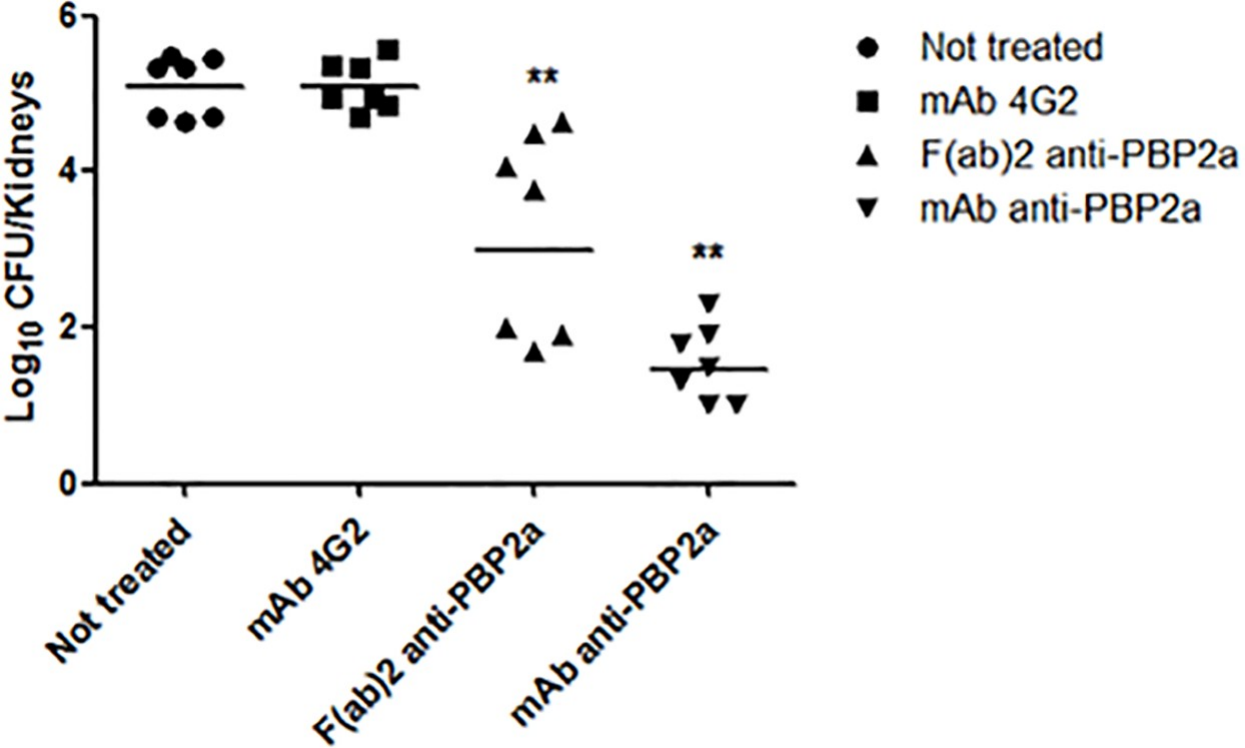
Bacterial load in kidneys of mice treated with MAb (IgG) and F(ab’)_2_ fragment. Mice (n = 7 mice/group) treated with MAb or F(ab’)_2_ anti-PBP2a fragment and controls (mAb 4G2 and not treated) were infected via IP administration of 2x10^8^ CFU of the BEC MRSA clone. Bacterial quantitation was performed 5 days later. Significant difference between treated and control groups was seen (*p<0.05). There was no significant difference between the control groups (untreated and mAb 4G2-treated).

There was a significant reduction in bacterial burden for the anti-PBP2a-fragment F(ab’)_2_-treated group when compared to that of both control groups (*p*<0.05); however, a major response was noted in the group treated with IgG MAb (Fig 2).

### Survival after challenge with a lethal dose of MRSA

**Fig 3:**
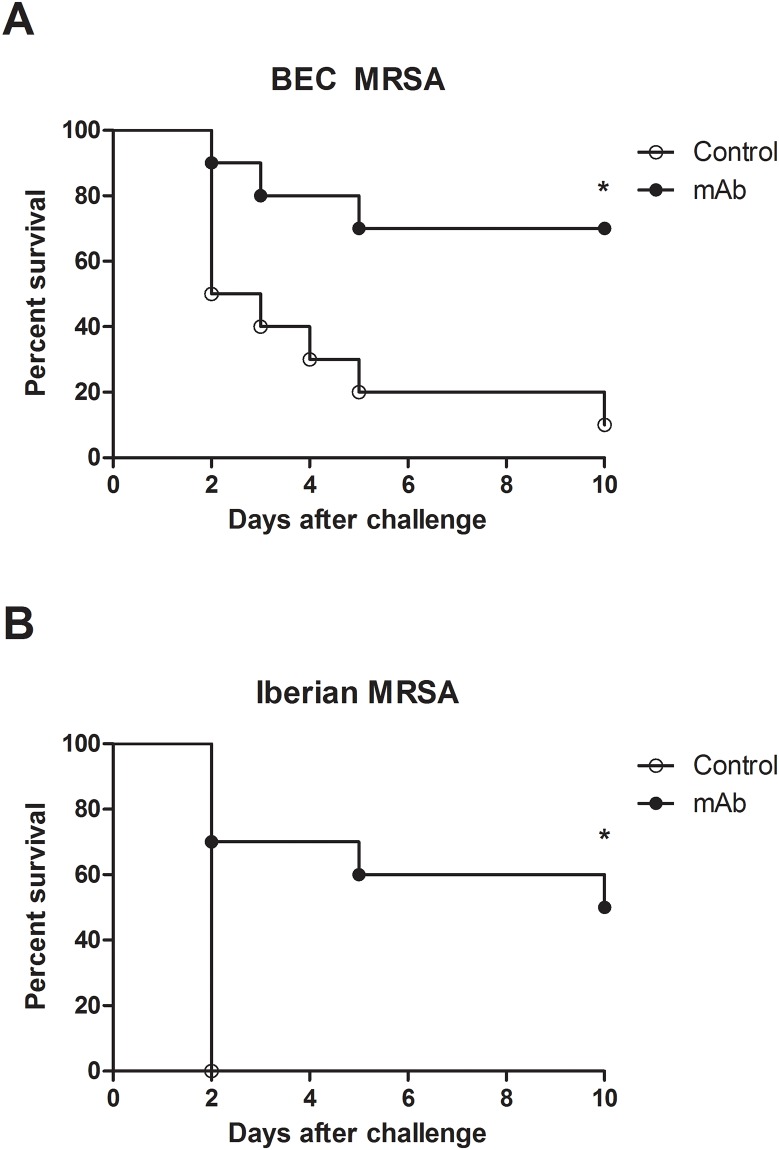
Effect of prophylactic treatment with anti-PBP2a MAb in a challenge with a lethal dose of BEC and Iberian MRSA clones. Previous treatment increased survival rates in the treated groups. Mice were challenged by IP inoculation with 4.2x10^8^ or 3.3x10^8^ CFU of the Iberian and BEC MRSA clones, respectively (*p<0.05).

To study the protection conferred by a previous treatment with anti-PBP2a MAb, the survival rate was determined after challenge with a bacterial load sufficient to kill more than 50% (5/10) of the animals. As shown in the Fig 3, for mice challenged with the BEC MRSA strain, 70% (7/10) of the animals survived the infection compared to only 10% (1/10) of the animals in the control group. Similarly, for mice challenged with the Iberian MRSA strain, 60% (6/10) of the treated animals survived, and 100% (10/10) died in the control group. Clinical symptoms were observed in treated and untreated animals. In addition, previous treatment with anti-PBP2a MAb prolonged survival. Differences observed in this assay could be due to a different bacteria inoculum and different levels of virulence among the MRSA strains used in this study. A second assay employing the Iberian MRSA strain was done and could be seen in Supporting information (S3 Fig).

### Therapeutic assay comparing vancomycin versus MAb treatment

**Fig 4:**
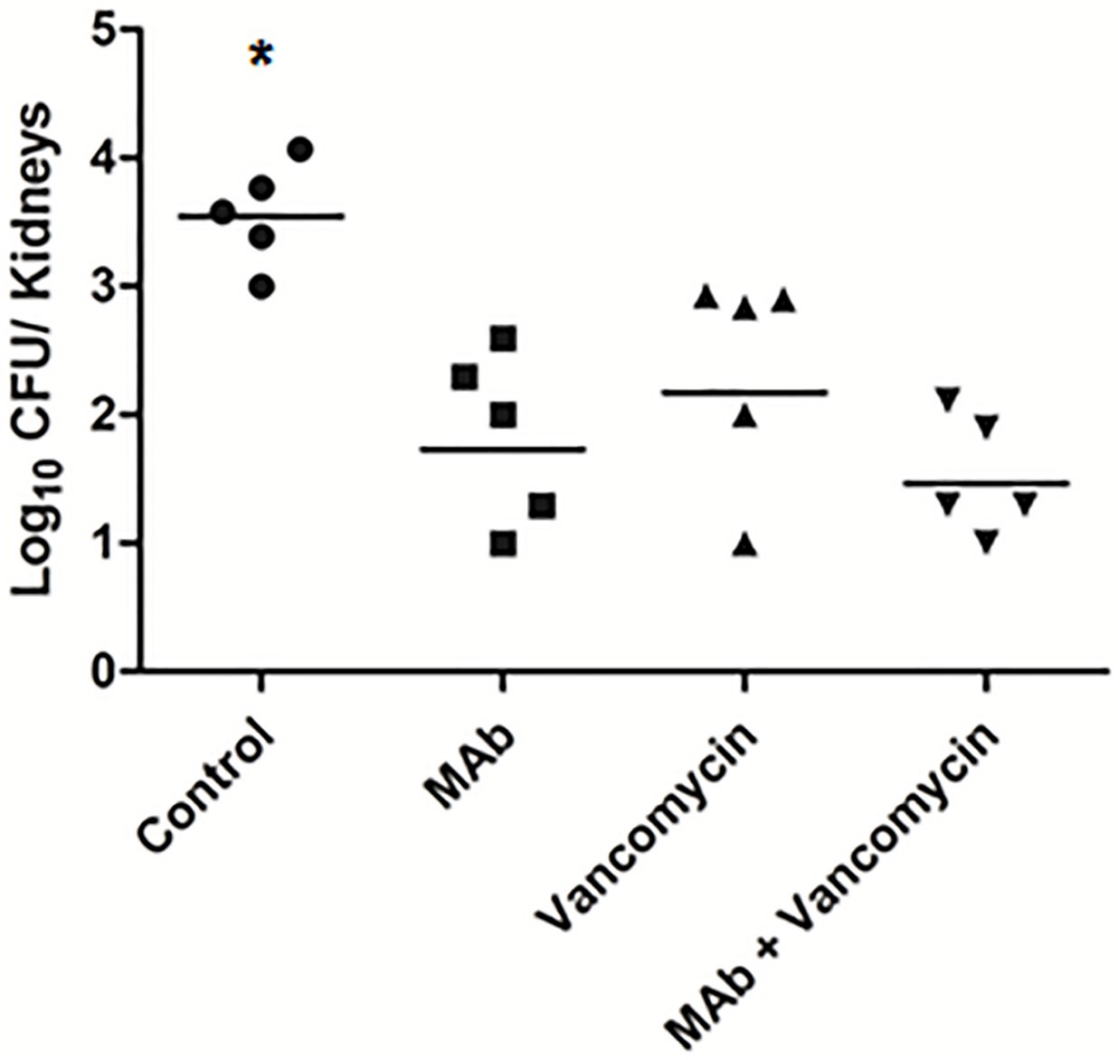
Effect of anti-PBP2a MAb and vancomycin monotherapy and MAb + vancomycin combination therapy. The bacterial load in kidneys are represented by group (five mice/group). Mice were infected with an IP inoculum of 7.0x10^6^ CFU of the BEC MRSA clone and then euthanized after 3 days. (*p<0.05 between the control group versus the other groups). The mean of recovered CFU by groups: Control group: 5020 CFU; Vancomycin-treated group: 336 CFU; MAb-treated group: 186 CFU; group treated with the MAb + vancomycin combination therapy: 52 CFU. The number of CFU/mice are represented in Supporting Information (S1 Table).

Since vancomycin is considered the first antimicrobial choice for the treatment of severe MRSA infections, a study was conducted using a therapeutic assay in which mice were infected and treated 6 h after infection. The vancomycin dose was adjusted and administered in a similar manner to that of the human treatment (500 mg every 12 h). These results showed that the treatment with MAb conferred a similar level of protection to that for the group treated with vancomycin (96.2% versus 93.4% bacterial load reduction, respectively). Furthermore, a slightly greater level of protection was observed for the group treated with both the antibiotic and antibody (98.9% bacterial load reduction) (Fig 4). A second assay yielded similar results (S4 Fig). Thus, the protection conferred by an antibody dose corresponded to five vancomycin doses, and the simultaneous administration of MAbs and vancomycin was also efficient for reducing bacterial load in the kidneys of infected animals.

### MAb biodistribution in tissues

**Fig 5:**
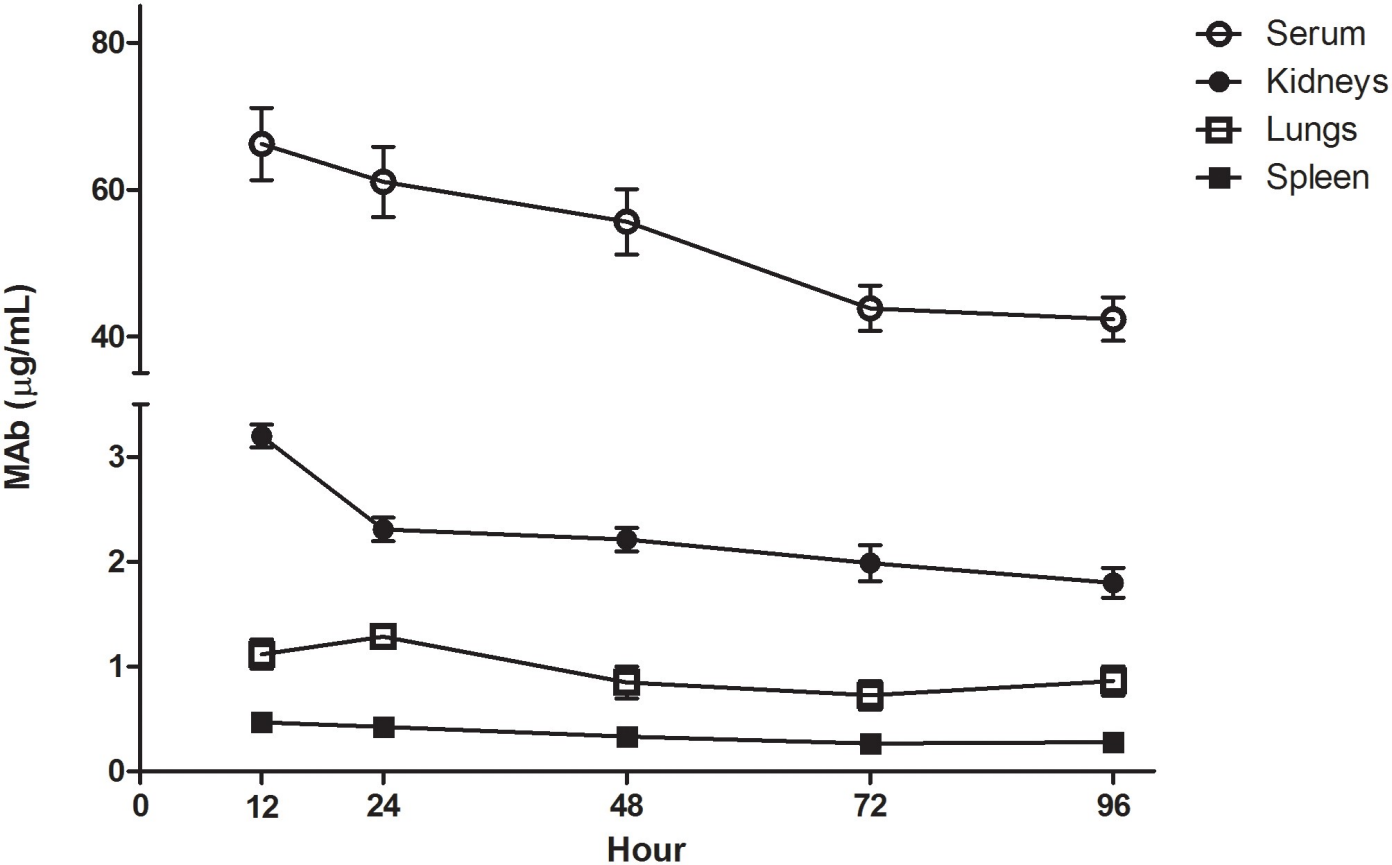
Biodistribution of the MAb *in vivo*. Serum, lungs, spleen, and kidneys of 9-week-old female BALB/c mice were obtained at different time points (12, 24, 48, 72, and 96 h) after IP injection of purified murine MAb (500 μg). Serum and tissues were processed and submitted for an ELISA to estimate the MAb concentration (μg/mL of serum or homogenated tissue) as described in the Material and methods. Control animal concentrations were estimated to be 0 (absorbance values were very close to those found at 0 μg of each respective standard curve, ranging from– 0.0044 to 0.0055). Results are shown as the mean ± S.E.M. of two measurements of three different animals for each timepoint.

Since staphylococcal infections can affect different tissues in the body, it is important to investigate whether a therapeutic molecule can penetrate different organs. ELISA was used to estimate the MAb concentration in tissues at different times. The results demonstrated that the MAb reached different tissues (Fig 5). Twelve hours after MAb administration, MAb concentrations in the serum, kidneys, lungs, and spleen were 66.2, 3.20, 1.12, and 0.47 μg/mL, respectively, and at 96 hours, they were 42.36, 1.80, 0.86, and 0.28 μg/mL, respectively. MAb concentrations in tissues were lower than that present in the serum (about 4.5% in the kidneys, 1.9% in the lungs, and 0.7% in the spleen). No clinical symptoms were observed in the animals used in these assays.

## Discussion

The emergence of infections caused by MDR bacteria is increasing at an alarming rate. This situation, in combination with a lack of options for treatment, makes this a worrying scenario. Of the common MDR bacteria, MRSA has few treatment options and it causes infections characterized by high morbidity. In this context, passive immunotherapy is a promising alternative, and it causes less toxicity, has an increased half-life (as an IgG), and, especially in our case, displays selective toxicity. These features could be considered distinct from conventional antibiotics. In order to prevent selective pressure, narrow-spectrum antimicrobial treatments are needed.

Previous studies of antibodies against PBP2a demonstrated no effects against methicillin-sensitive *S*. *aureus* [11], confirming the specificity of anti-PBP2a antibodies against MRSA. The MAb presented here was derived from this work, and it was obtained and characterized in previous studies [11,13,14]. We previously demonstrated that this MAb binds strongly to recombinant PBP2a and recognizes native PBP2a protein on the bacteria. As shown in the S5 Fig, a region of 88 amino acids comprising the active center (serine protease motif—STQK) was used in the immunization of mice to generate this monoclonal antibody. This region is surface-exposed, and we hypothesize that the MAb mechanism of action involves interference with the enzymatic activity of PBP2a. In the present work, we evaluated the protection conferred by this MAb in an animal model, which included the use of assays to show bacterial load reduction in the kidneys, survival after challenge with a lethal dose, and a comparison with vancomycin treatment in a therapeutic assay.

The epidemiology of MRSA infections has revealed the presence of the predominant clonal types responsible for outbreaks and epidemics in hospitals worldwide. These clones have a higher colonization and virulence capacity when compared to that of non-epidemic MRSA strains [18]. We chose to work with these strains because Brazilian epidemics and Iberian MRSA clones are the predominant strains isolated from patients infected with MRSA in South America and Europe. We expect that this antibody confers protection against other MRSA strains for the following reasons: (i) the protection generated by administration of the anti-PBP2a MAb for both epidemic clones and (ii) the high degree of conservation in the PBP2a amino acid sequences of different MRSA strains.

We evaluated the extent of protection conferred by MAb treatment using a prophylactic assay in a murine model. Previous treatment with anti-PBP2a MAb resulted in significant bacterial load reduction in the kidneys of mice, in addition to reduced mortality and prolonged survival after bacterial challenge. These results are in accordance with previous studies of DNA vaccination using the *mec*A gene [11] and a recent report using a passive immunization approach [19], demonstrating that antibodies directed against PBP2a can confer protection against MRSA infections in animal models. The protection observed in animals treated with the antibody devoid of the Fc region (F[ab’]_2_ fragment) means that the antibody can act independently of the effector functions of the host immune system. Since several MRSA-infected patients can be immunosuppressed, neutralizing activity would be considered an important feature of a successful treatment for these infections.

MAbs and antibiotics display different structures, sizes, mechanisms of action, and pharmacokinetics. The MAb biodistribution results have shown that most of the antibody remains in the serum, and only a small proportion reaches the tissues. These results are in accordance with other studies [20]. These characteristics could be useful for treating patients at the onset of a MRSA infection. Since MRSA infections cause high morbidity, treatment must start quickly, and combining multiple drugs may be more efficient than monotherapies. Due to their small molecular weights, antibiotics reach tissues more easily than IgG MAbs.

Peptidoglycan biosynthesis is an essential prerequisite for bacterial viability. Since this macromolecule is found exclusively in bacteria, it is considered an excellent target. Vancomycin act by binding the D-Ala-D-Ala terminus of nascent peptidoglycan pentapeptides, and this antibody binds the transpeptidase region, which likely impairs the enzymatic activity of PBP2a. The association between an antibiotic and a specific antibody acting in the same environment resulted in an efficient reduction in the bacterial load in therapeutic assay. A few days after treatment, the number of bacteria in the kidneys was drastically reduced. Although combined treatments were not shown to be statistically better than single treatments in our study, a small difference in bacterial clearance could provide better therapeutic outcomes.

Since IgG MAbs usually have an extended half-life [20], we can expect that this MAb could be used to treat systemic infections caused by MRSA. It could also be used in pre-operative cases that have a high risk for MRSA infections. In the future, we intend to evaluate this MAb in combination with other anti-staphylococcal antibiotics, such as linezolid, daptomycin, and tigecyclin.

## Conclusion

In summary, we have demonstrated that an anti-PBP2a MAb confers protection against MRSA infections in a murine model. Furthermore, the combination of this MAb with vancomycin yields greater protection than a MAb or vancomycin monotherapy alone.

## Supporting information

S1 File NC3Rs ARRIVE guidelines D19-10773. (PDF)

S1 Table Number of CFUs recovered from mice used in the therapeutic assay (Fig 4). (DOCX)

S1 Fig A gel resulting from non-reducing and reducing SDS-PAGE showing the purified MAb anti-PBP2a. Lanes 1 and 14 MW molecular weight marker. Lane 2 to 6: non-reducing samples, showing a protein with approximately 150 kDa. Lane 7 to 12: reducing samples, showing two proteins with 50 and 25 kDa, corresponding to the heavy and light chain, respectively.
(TIF)

S2 Fig Schematic representation of the timeline for all experiments performed in this study. The timeline is represented in days (A to D) and in hours (E). **A**. Lethal and sublethal dose determination; **B**. Systemic infection and bacterial renal quantification assays; **C**. Survival assay; **D**. Therapeutic assay with vancomycin; **E**. Biodistibution assay. VMC: Vancomycin.
(TIF)

S3 Fig Effect of prophylactic treatment with anti-PBP2a MAb in a challenge with a lethal dose of Iberian MRSA clone. Previous treatment increased survival rates in the treated group. Mice were challenged by IP inoculation with 6.5x10^8^ CFU of the Iberian MRSA clone (*p<0.05).
(TIF)

S4 Fig Effect of treatment with anti-PBP2a MAb, vancomycin, and the combination therapy (MAb + vancomycin) in a therapeutic assay (II). The bacterial load in the kidneys is represented by groups (n = 5 mice/group). Mice were infected with an IP inoculum of 6.0x10^7^ CFU of the BEC MRSA clone and euthanized 3 days later (*p<0.05 for comparison between the control group versus the vancomycin- and MAb-treated groups). The mean for the recovered CFU by groups: Control group: 270,300 CFU; Vancomycin treated group: 12,020 CFU; MAB treated group: 12,344 CFU; MAB + Vancomycin-treated group: 4,490 CFU.
(TIFF)

S5 Fig Representation of the PBP2a molecule. The structure was obtained from Protein Data Bank (PDB; ID 1MWT). A surface representation of the whole molecule is shown in blue. The 88-amino acid region used to generate the anti-PBP2a MAb is marked in red (surface/ribbon). In yellow, amino acids comprising the active center are shown (serine protease motif—STQK).
(TIF)

## References

[1] O’Neil J. Review on antibiotic resistance. Tackling drug resistance globally. 2016; WHO.

[2] Ryffel C, Strassle A, Kayser FH, Berger-Bächi B. Mechanisms of heteroresistance in methicillin-resistant *Staphylococcus aureus*. *Antimicrob Agents Chemother* 1994; 38:724–728. 10.1128/aac.38.4.724 8031036 PMC284532

[3] Gardete S, Tomasz A. Mechanisms of vancomycin resistance in *Staphylococcus aureus*. *J Clin Invest*. 2014;124:2836–2840. 10.1172/JCI68834 24983424 PMC4071404

[4] Casadevall A. The third age of antimicrobial therapy. *Clin Infect Dis*. 2006; 42:1414–1416. 10.1086/503431 16619153

[5] DiGiandomenico A, Sellman BR. Antibacterial monoclonal antibodies: The next generation? *Curr Op in Microbiol*. 2015; 27:78–85. 10.1016/j.mib.2015.07.014 26302478

[6] Weisman LE, Thackray HM, Garcia-Prats JA, Nesin M, Schneider JH, Fretz J, et al Phase 1/2 double-bind, placebo controlled, dose escalation, safety and pharmacokinetic study of pagibaxiMAb, an antistaphylococcal monoclonal antibody for the prevention of staphylococcal bloodstream infections, in very-low-birth-weight neonates. *Antimicrob Agents and Chemother*. 2009; 53:2879–2886. 19380597 10.1128/AAC.01565-08 PMC2704668

[7] Ragle BE, Bubeck WJ. Anti-alpha-hemolysin monoclonal antibodies mediate protection against *Staphylococcus aureus* pneumonia. *Infect Imm*. 2009; 77:2712–2718. 10.1128/IAI.00115-09 PMC2708543 19380475

[8] Hall AE, Domanski PJ, Patel PR, Vernachio JH, Syribeys PJ, Gorovits EL, et al Characterization of a protective monoclonal antibody recognizing *Staphylococcus aureus* MSCRAMM protein clumping factor A. *Infect Imm*. 2003; 71:6864–6870. 10.1128/IAI.71.12.6864-6870.2003 PMC308922 14638774

[9] Kim HK, Cheng AG, Kim HY, Missiakas DM and Schneewind O. Non toxigenic protein A vaccine for methicillin-resistant *Staphylococcus aureus* infections in mice. *J Exp Med*. 2010; 207(9):1863–1870. 10.1084/jem.20092514 20713595 PMC2931167

[10] Goffin C, Ghuysen JM. Multimodular penicillin binding proteins: An enigmatic family of orthologs and paralogs. *Microbiol Mol Biol Rev*. 1998; 62:1079–1093. 9841666 10.1128/mmbr.62.4.1079-1093.1998 PMC98940

[11] Senna JP, Roth DM, Oliveira JS, Machado DC, Santos DS. Protective immune response against methicillin resistant *Staphylococcus aureus* in a murine model using a DNA vaccine approach. *Vaccine*. 2003; 21:2661–2666. 10.1016/s0264-410x(02)00738-7 12744903

[12] Ohwada A, Sekiya M, Hanaki H, Arai KK, Nagaoka I, Hori S, et al. DNA vaccination by *mec*A sequence evokes an antibacterial immune response against methicillin-resistant *Staphylococcus aureus*. *J Antimicrob Chemother*. 1999; 44:767–774. 10.1093/jac/44.6.767 10590277

[13] Senna JP, Teixeira M da G, Santiago M de A, Batoréu NM, Valadares N, Galler R. Generation and characterization of murine monoclonal antibodies anti-PBP2a of methicillin-resistant *Staphylococcus aureus*. *Monoclon Antib Imunodiagn Immunother*. 2015; 34:257–262. 10.1089/mab.2015.0013 26301929

[14] Araujo AEV., Souza NP, Souza APB, Senna JPM. Production and characterization of F(Ab’)2 fragments obtained by enzymatic digestion from murine anti-MRSA PBP2a monoclonal antibodies. *Appl Biochem Biotechnol*. 2016; 185:72–80. 10.1007/s12010-017-2624-z 29082478

[15] Enright MC, Robinson DA, Randle G, Feil EJ, Grundman H and Spratt BG. The evolutionary history of methicillin-resistant *Staphylococcus aureus* (MRSA). *PNAS*. 2002 99(11): 7687–7692. 10.1073/pnas.122108599 12032344 PMC124322

[16] Costa MO, Beltrame CO, Ferreira FA, Botelho AM, Lima NC, Souza RC, et al Complete genome sequence of a variant of the methicillin-resistant *Staphylococcus aureus* ST239 lineage, strain BMB9393, displaying superior ability to accumulate ica-independent biofilm. *Genome Announc*. 2013; 1:pii: e00576–13. 10.1128/genomeA.00576-13 PMC3738891 23929475

[17] Araujo AEV, dos Santos IB, Freire IM, Bonin RF, Machado LA, Senna JPM. Determination of lethal and sublethal doses of *Acinetobacter baumannii* and Methicillin-resistant *Staphylococcus aureus* (MRSA) in murine models using a reduced number of animals. *J Exp Appl Animal Sci*. 2015; 3:336–340.

[18] Papakyriacou H, Vaz D, Simor A, Louie M, McGavin MJ. Molecular analysis of the accessory gene regulator (*agr*) locus and balance of virulence factor expression in epidemic methicillin-resistant *Staphylococcus aureus*. *J Infec Dis*. 2000; 181:2400–2404. 10.1086/315342 10720522

[19] Naghshbandi RZ, Haghighat S, Mahdavi M. Passive immunization against methicillin resistant *Staphylococcus aureus* recombinant PBP2a in sepsis model of mice: Comparable results with antibiotic therapy. *Int Immunopharmacol*. 2018; 56:186–192. 10.1016/j.intimp.2018.01.035 29414649

[20] Shah DK, Betts AM. Antibody biodistribution coefficients: inferring tissue concentrations of monoclonal antibodies bases on the plasma concentrations in several preclinical species and human. 2013; MAbs 5:297–305. 10.4161/mabs.23684 23406896 PMC3893240

