# Supplementary material for: Monoclonal antibody anti-PBP2a protects mice against MRSA (methicillin-resistant Staphylococcus aureus) infections
Source: PLoS One. 2019 Nov 27;14(11):e0225752. doi: 10.1371/journal.pone.0225752 (PMC6880988; doi:10.1371/journal.pone.0225752)
Supplement: S1 Table — (DOCX) [file pone.0225752.s002.docx]

SI Table. Number of CFUs recovered from each mice in the therapeutic assay (Fig 4).

| Control | Mab treated | Vancomycin treated | Association |
| --- | --- | --- | --- |
| 118.000 | 600 | 860 | 130 |
| 6.000 | 200 | 700 | 80 |
| 3.800 | 100 | 100 | 20 |
| 2.500 | 20 | 10 | 20 |
| 1.000 | 10 | 10 | 10 |
