# Supplementary figures and images for: Monoclonal antibody anti-PBP2a protects mice against MRSA (methicillin-resistant Staphylococcus aureus) infections
Source: PLoS One. 2019 Nov 27;14(11):e0225752. doi: 10.1371/journal.pone.0225752 (PMC6880988; doi:10.1371/journal.pone.0225752)

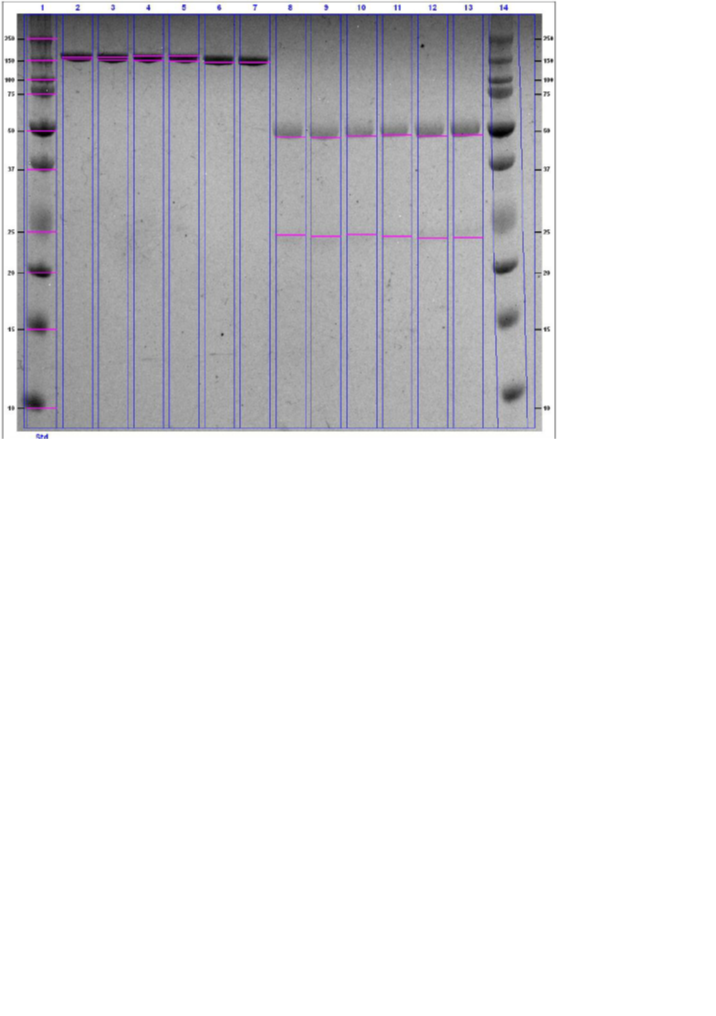

Supplement: S1 Fig — Lanes 1 and 14 MW molecular weight marker. Lane 2 to 6: non-reducing samples, showing a protein with approximately 150 kDa. Lane 7 to 12: reducing samples, showing two proteins with 50 and 25 kDa, corresponding to the heavy and light chain, respectively. (TIF) [file pone.0225752.s003.tif]

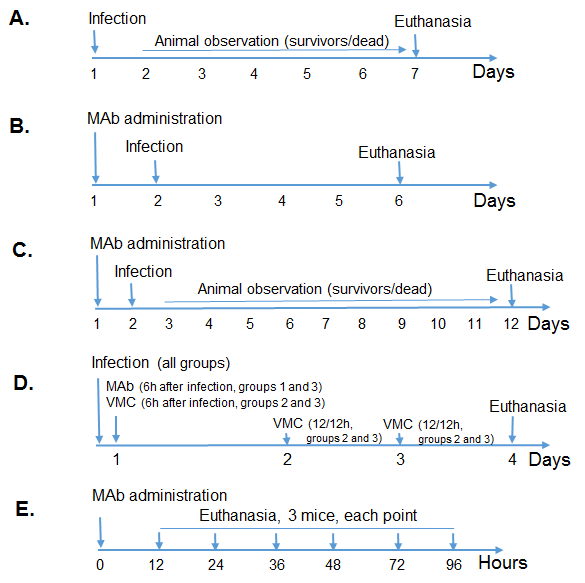

Supplement: S2 Fig — The timeline is represented in days (A to D) and in hours (E). A. Lethal and sublethal dose determination; B. Systemic infection and bacterial renal quantification assays; C. Survival assay; D. Therapeutic assay with vancomycin; E. Biodistibution assay. VMC: Vancomycin. (TIF) [file pone.0225752.s004.tif]

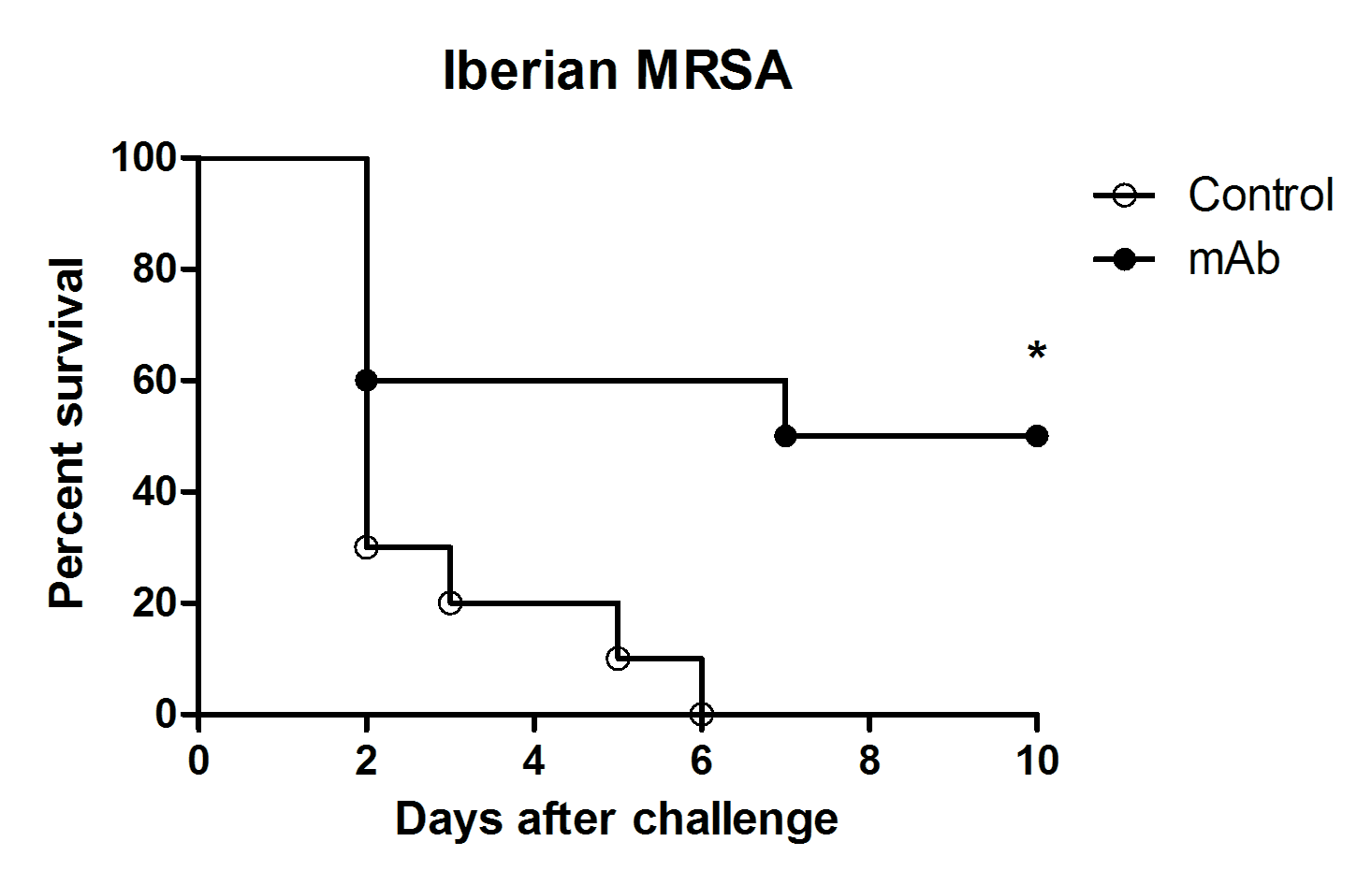

Supplement: S3 Fig — Previous treatment increased survival rates in the treated group. Mice were challenged by IP inoculation with 6.5x10^8^ CFU of the Iberian MRSA clone (*p<0.05). (TIF) [file pone.0225752.s005.tif]

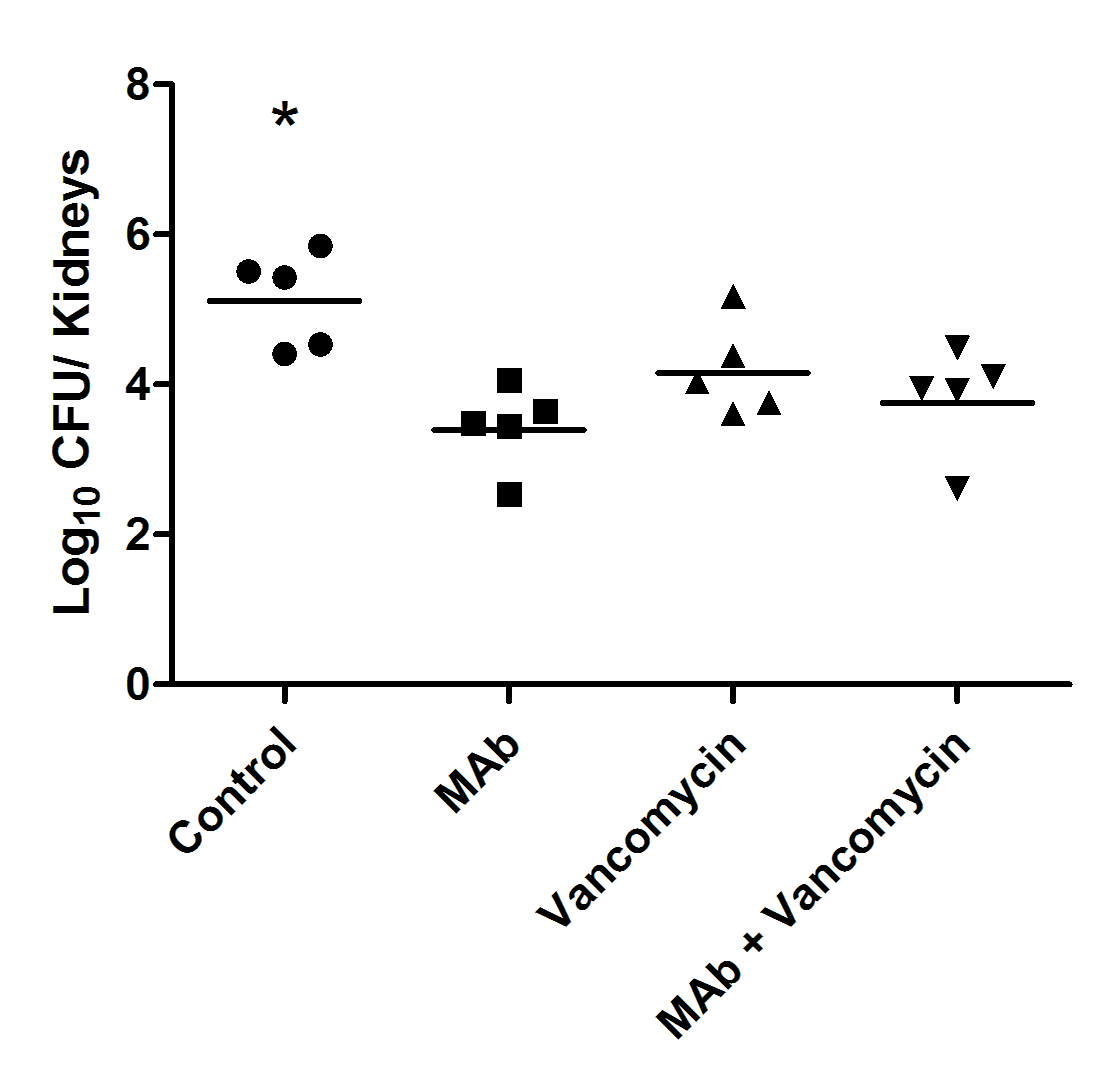

Supplement: S4 Fig — The bacterial load in the kidneys is represented by groups (n = 5 mice/group). Mice were infected with an IP inoculum of 6.0x10^7^ CFU of the BEC MRSA clone and euthanized 3 days later (*p<0.05 for comparison between the control group versus the vancomycin- and MAb-treated groups). The mean for the recovered CFU by groups: Control group: 270,300 CFU; Vancomycin treated group: 12,020 CFU; MAB treated group: 12,344 CFU; MAB + Vancomycin-treated group: 4,490 CFU. (TIFF) [file pone.0225752.s006.tiff]

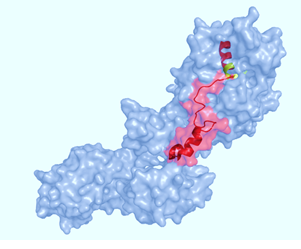

Supplement: S5 Fig — The structure was obtained from Protein Data Bank (PDB; ID 1MWT). A surface representation of the whole molecule is shown in blue. The 88-amino acid region used to generate the anti-PBP2a MAb is marked in red (surface/ribbon). In yellow, amino acids comprising the active center are shown (serine protease motif—STQK). (TIF) [file pone.0225752.s007.tif]
